# Intermediate DNA methylation is a conserved signature of genome regulation

**DOI:** 10.1038/ncomms7363

**Published:** 2015-02-18

**Authors:** GiNell Elliott, Chibo Hong, Xiaoyun Xing, Xin Zhou, Daofeng Li, Cristian Coarfa, Robert J.A. Bell, Cecile L. Maire, Keith L. Ligon, Mahvash Sigaroudinia, Philippe Gascard, Thea D. Tlsty, R. Alan Harris, Leonard C. Schalkwyk, Misha Bilenky, Jonathan Mill, Peggy J. Farnham, Manolis Kellis, Marco A. Marra, Aleksandar Milosavljevic, Martin Hirst, Gary D. Stormo, Ting Wang, Joseph F. Costello

**Affiliations:** 1 Department of Genetics, Center for Genome Sciences and Systems Biology, Washington University School of Medicine, St Louis, Missouri 63108, USA; 2 Brain Tumor Research Center, Department of Neurosurgery, Helen Diller Family Comprehensive Cancer Center, University of California, San Francisco, San Francisco, California 94115, USA; 3 Department of Molecular and Human Genetics, Baylor College of Medicine, Houston, Texas 77030, USA; 4 Institute for Human Genetics, University of California San Francisco, San Francisco, California 94158, USA; 5 Department of Medical Oncology, Center for Molecular Oncologic Pathology, Dana-Farber Cancer Institute, Boston, Massachusetts 02115, USA; 6 Department of Pathology, Helen Diller Family Comprehensive Cancer Center, University of California, San Francisco, San Francisco, California 94115, USA; 7 School of Biological Sciences, University of Essex, Colchester CO4 3SQ, UK; 8 Canada's Michael Smith Genome Sciences Centre, BC Cancer Agency, Vancouver, British Columbia V5Z 1L3, Canada; 9 Social Genetic & Developmental Psychiatry Centre, Institute of Psychiatry, King’s College London, London WC2R 2LS, UK; 10 University of Exeter Medical School, Exeter University, St Luke's Campus, Exeter EX1 2LU, UK; 11 Department of Pharmacology and the Genome Center, University of California-Davis, Davis, California 95616, USA; 12 The Broad Institute of MIT and Harvard, Cambridge, Massachusetts 02142, USA; 13 Centre for High-Throughput Biology and Department of Microbiology & Immunology, University of British Columbia, Vancouver, British Columbia, Canada V6T 1Z4

## Abstract

The role of intermediate methylation states in DNA is unclear. Here, to comprehensively identify regions of intermediate methylation and their quantitative relationship with gene activity, we apply integrative and comparative epigenomics to 25 human primary cell and tissue samples. We report 18,452 intermediate methylation regions located near 36% of genes and enriched at enhancers, exons and DNase I hypersensitivity sites. Intermediate methylation regions average 57% methylation, are predominantly allele-independent and are conserved across individuals and between mouse and human, suggesting a conserved function. These regions have an intermediate level of active chromatin marks and their associated genes have intermediate transcriptional activity. Exonic intermediate methylation correlates with exon inclusion at a level between that of fully methylated and unmethylated exons, highlighting gene context-dependent functions. We conclude that intermediate DNA methylation is a conserved signature of gene regulation and exon usage.

## Abstract (web summary)

Many loci in the mammalian genome are intermediately methylated. Here, by comprehensively identifying these loci and quantifying their relationship with gene activity, the authors show that intermediate methylation is an evolutionarily conserved epigenomic signature of gene regulation.

## Graphical abstract

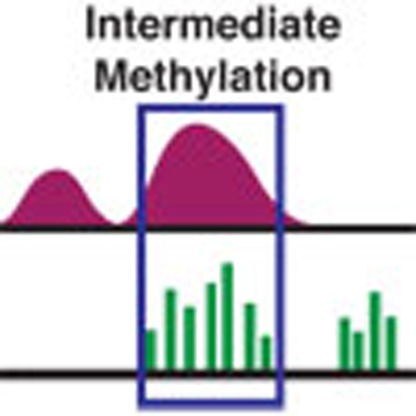

DNA methylation plays a central role in human development and cellular identity^1^,^2^,^3^,^4^,^5^,^6^. Dynamic CpG methylation throughout cell differentiation correlates with cell type-specific gene regulation and expression levels, with loss of methylation reflecting enhancer or gene activation^7^,^8^,^9^,^10^. The majority of CpGs in differentiated cells are uniformly methylated or unmethylated between homologous chromosomes and within cell populations composed of a single cell type. The bimodality of DNA methylation implies a binary on–off control over gene expression, yet a significant number of loci throughout the genome do not fit within this model.

Regions of intermediate methylation (IM) may exhibit allelic methylation, intercellular variability or clusters of interspersed methylated and unmethylated CpGs within each cell. A small number of genes are marked by allele-specific methylation (ASM), including imprinting control regions (ICRs), which are essential for mammalian development^11^,^12^,^13^,^14^,^15^,^16^,^17^. Other IM states, including non-allelic IM, may also be functional and potentially dynamically regulated during development^7^,^18^,^19^. The level of methylation reported in IM regions in prior studies is variable, but typically much less than the 50% that typifies ICRs^7^,^18^,^19^. The prevalence of IM states and their function within genetically and phenotypically homogenous cell populations has been enigmatic.

Here, we use genome-wide DNA methylation profiling to identify and compare IM regions across human tissues and primary cell types, and among individuals. We define boundaries of IM loci by combining two independent and complementary enrichment-based methods that, respectively, identify methylated and unmethylated regions of DNA. We then validate and quantify methylation levels at basepair resolution within IM regions using whole-genome bisulfite sequencing (WGBS) and methylation array data. We analyse multiple cell types from the same individual to distinguish cell type epigenetic differences from genetic effects on the epigenome. We then examine the relationship between IM states, associated histone marks of gene regulation, mRNA levels and exon usage from the same samples used to discover the IM regions. We further use these data, along with *in vivo* enhancer assay data and evolutionary conservation, to discover potential functions of IM states.

## Results

### IM is closely associated with genes

To identify regions of IM, we combined two complementary whole-methylome profiling methods: MeDIP-Seq, which targets methylated DNA using an anti-methylcytosine antibody; and MRE-Seq, which identifies unmethylated DNA by methylation-sensitive restriction enzyme digestions. This experimental approach was chosen because nearly all mapped reads are informative, contrasting to the inefficiency of WGBS in which 70–80% of sequence reads are uninformative because they lack CpG sites^10^. Furthermore, 5-methylcytosine and 5-hydroxymethylcytosine are conflated in WGBS, whereas MeDIP/MRE-Seq detect 5-methylcytosine exclusively. MeDIP/MRE-Seq may also provide greater accuracy in a minority of loci^20^. Most importantly, identification of IM with MeDIP/MRE-Seq does not assume a binary state, as IM are identified by strong and overlapping signals from each assay. We applied MeDIP/MRE-Seq^8^,^20^,^21^,^22^,^23^ to 23 human primary cell and tissue samples isolated without culturing from three tissue types and seven donors^24^, and two biological replicates of the H1 embryonic stem cell line (H1ES)^23^ (see Methods). The diverse set of samples we profiled allowed controlled comparisons of different cell types from the same individual, and identical cell types from different age-matched donors.

**Figure 1:**
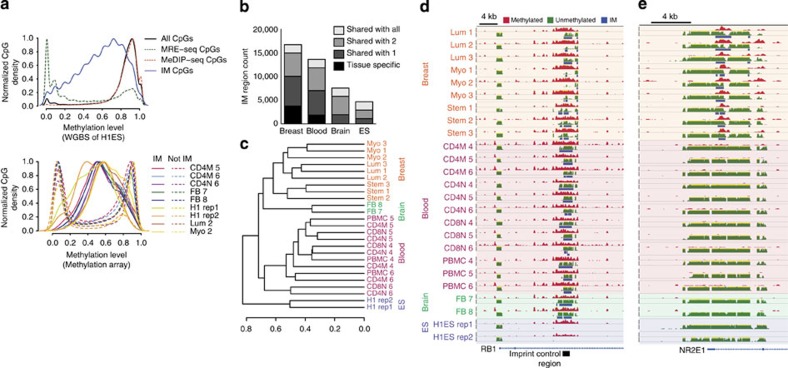
IM is predominantly tissue specific. (**a**) Top panel, comparison of WGBS methylation levels at CpGs carrying only MRE-seq or MeDIP-seq reads, and CpGs within IM regions. Bottom panel, comparison of 450k Infinium array methylation levels at CpGs in IM regions and outside of IM regions (66% of all IM regions overlap one or more methylation array probes). A value of 0 is unmethylated, a value of 1 is fully methylated. (**b**) Comparison of the number of IM regions specific to one or more of the four tissues studied. (**c**) Hierarchical clustering of cell type similarity based on the presence or absence of IM status. Distance metric is Jaccard; clustering method is average. (**d**) The known imprinted locus in the body of the Rb gene was detected as IM in all tissues except ES cells using MeDIP-seq/MRE-seq. (**e**) A breast-specific IM region. (**d**,**e**) Height for all tracks shows a signal range from 0 to 50 reads.

A small fraction of CpGs in each sample showed signal enrichment in both MeDIP-Seq and MRE-Seq, indicating simultaneous presence of methylated and unmethylated DNA (Fig. 1a and Supplementary Fig. 1a). We developed a maximum scoring segment algorithm to define boundaries of IM regions representing clusters of neighbouring CpGs with co-occurring enrichment in MeDIP/MRE-Seq (Methods; Supplementary Fig. 1b). Altogether, 18,452 unique autosomal IM regions were detected in one or more samples, containing 2% of the 26.9 million CpGs interrogated. Within IM regions, we estimated the methylation levels at single CpGs using WGBS from H1ES cells and Illumina 450K Infinium Methylation Array data from eight of our samples. WGBS and methylation arrays confirmed the primarily bimodal genome-wide distribution of fully methylated and unmethylated CpGs. Individual CpGs within regions classified as IM, however, had a mean methylation of 57% (WGBS) and 58% (arrays; Fig. 1a). In the majority of regions, the IM state was restricted to one or a subset of tissue or cell types, although 1,754, or 9%, were present across all tissues studied (constitutive IM; Fig. 1b–e). Hierarchical clustering based on the presence or absence of the IM status or based on MeDIP/MRE-Seq read density at the union set of IM regions strongly separated cell types isolated from different tissues (Fig. 1c and Supplementary Fig. 2).

**Figure 2:**
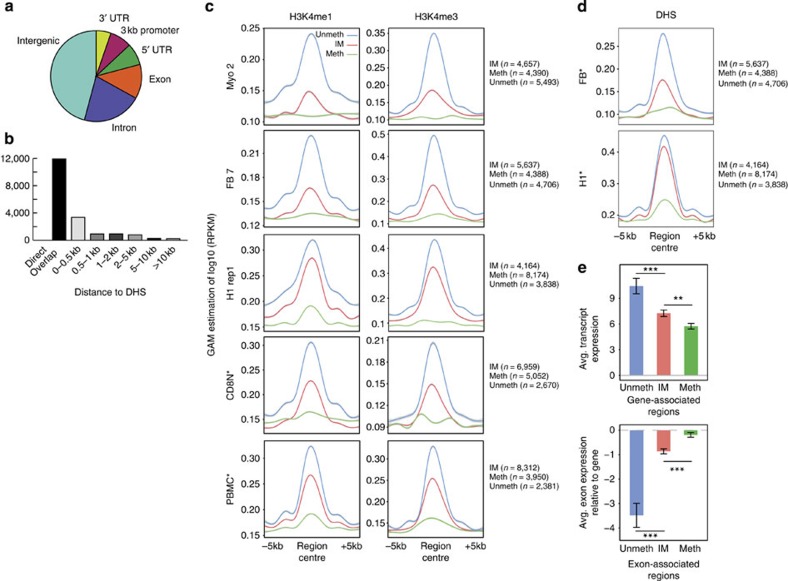
IM is associated with intermediate levels of epigenomic modifications and gene expression. (**a**) Distribution of IM CpGs over Refseq genome feature annotations. (**b**) Distance from each IM region to the nearest DHS (*P*<0.001, *χ*^2^; odds ratio=2.53). DHSs were compiled from 41 different cell types, covering approximately 8% of the genome. (**c**) Comparison of H3K4me3 and H3K4me1 signals between methylated (Meth), unmethylated (Unmeth) and IM regions in five different cell types using a generalized additive model (GAM; grey outlines indicate 95% confidence interval (CI); *donor for histone ChIP-seq does not match donor used in IM analysis). (**d**) Comparison of DHS signals between Meth, Unmeth and IM regions (grey outlines indicate 95% CI; *donor for DNase-seq does not match donor used in IM analysis). (**e**) Top panel, average whole-transcript expression of genes associated with Meth, Unmeth and IM regions. Bottom panel, average exon expression relative to its gene expression for exons within 1 kb of Meth, Unmeth and IM regions (error bars represent s.e.m.; **P<0.005, Wilcoxon; ***P<0.0001, Wilcoxon). Expression analysis was based on mRNA-seq data from breast myoepithelial cells. Total transcript-associated regions: IM=6,776; Meth=3,270; Unmeth=5,605. Total exon-associated regions: IM=14,336; Meth=6,642; Unmeth=9,331.

We detected an average of 7,531 autosomal IM regions per sample, with a mean length of 271 bp. We selected the union of IM regions across all samples (*n*=18,452) as a reference set for further analysis. Over half of IM regions were intragenic or were within 3 kb of 6,798 autosomal genes (36% of genes; odds ratio=1.27; *P*-value<0.001, *χ*^2^). IM regions were enriched at promoters, exons and DNase I hypersensitivity sites (DHSs), and confirmed 17 of 19 known imprinted loci (Fig. 2a,b and Supplementary Figs 2a and 3). Thus, IM regions were associated with a significant number of genes and potential regulatory regions.

Approximately 50% of IM regions overlapped loci identified as differentially methylated across cell and tissue types^10^, consistent with our observation that IM is often tissue-specific. However, most studies comparing methylation between cell types examine only the binary direction of the methylation change (hypo- or hypermethylated) rather than the methylation level maintained within each cell type. Thus, differential methylation as previously defined does not identify specific regions as IM. The majority (69%) of IM regions reported here have not been previously identified as having allele-specific or stable IM states within a population of cells of a single type^7^,^17^,^18^,^25^,^26^ (Supplementary Fig. 5a,b).

### Level of histone modification and DHS at IM

To determine whether IM regions could have regulatory function, we next performed chromatin immunoprecipitation sequencing (ChIP-Seq) for selected histone modifications that demarcate active regulatory elements, using the same cell samples in which IM regions were mapped. Within a given sample, we explored the relationship between DNA methylation level and histone modification levels by comparing histone ChIP-Seq signal at IM regions to signal at regions that were methylated and unmethylated in the same sample used for ChIP-Seq, but which had IM status in other samples. Consistent with prior studies, the normalized read densities for the active marks of H3K4me1 and H3K4me3 were anti-correlated with DNA methylation. Interestingly, signal strength for these modifications at regions with IM status consistently fell between signals at methylated and unmethylated sites, providing a novel association of IM with intermediately active chromatin states (Fig. 2c). Furthermore, DHS in H1ES cells or in fetal brain had a similar intermediate state at their IM regions, respectively (Fig. 2d).

Enhancer regions predicted by chromHMM^27^ using the cell-type-matched ChIP-Seq data were more enriched for IM CpGs than the genomic background of methylated (MeDIP-Seq only) or unmethylated (MRE-Seq only) CpGs (Supplementary Methods and Supplementary Fig. 3). We therefore asked if IM regions coincide with functional regulatory elements defined by *in vivo* transgenic reporter assays. Of 70 candidate enhancers defined by the VISTA project that we also identified as IM regions, 50 drove transgene expression in mouse embryos^28^—a higher validation rate than VISTA candidates tested from non-IM regions. Methylation levels of enhancers in the reporter construct are unknown, however, this result demonstrates that many IM regions coincide with functional enhancers (Supplementary Data 1). A small portion of IM regions (9.5%) in fetal brain also shows increased 5 hmC in fetal brain relative to adult brain^29^ (Supplementary Fig. 5c).

### Level of gene expression and exon inclusion near IM

We hypothesized that the DNA methylation level in IM regions might correspond to a difference in the level of transcription of the associated gene. To examine this relationship, we initially focused on IM regions occurring within 10 kb of transcription start sites in breast myoepithelial cells. For comparison, we applied the same selection criteria to methylated and fully unmethylated regions in myoepithelial cells that had IM status in other cell types. Methylated and unmethylated status at these regions distinguished proximal genes with significantly different mean expression values, following the established inverse correlation between DNA methylation at enhancers and gene expression. Remarkably, despite an average DNA methylation level of 63% in myoepithelial cells based on methylation array, regions with IM status corresponded to a set of genes with mean expression distinct from both the methylated and unmethylated sets (*P*<0.005, Wilcoxon), suggesting that enhancers with IM are associated with intermediate levels of gene expression (Fig. 2e).

IM regions were dramatically enriched at coding exons (Supplementary Methods and Supplementary Fig. 3). Exons associated with IM were defined as exons with at least one IM region in the exon or within 1 kb of the exon boundaries. To investigate the potential function of exonic IM, we calculated expression of each exon relative to the expression of its gene as a measure of exon inclusion in the transcript, and compared this relative expression of IM exons to that of methylated or unmethylated exon-associated regions. Interestingly, the relationship between methylation and relative exon expression was the opposite of that observed between expression and methylation at candidate enhancers. Methylated regions were associated with the highest exon inclusion, whereas unmethylated regions showed the lowest exon inclusion, IM states being associated with intermediate inclusion (*P*<0.001, Wilcoxon; Fig. 2e). These results were consistent with previous reports using binary classification of DNA methylation states^30^ and extended the relationship specifically to the IM state. Therefore, the level of DNA methylation could potentially affect either gene expression or exon inclusion rates in a more quantitative manner than previously appreciated, although functional assays will be needed.

### IM is predominantly allele independent

**Figure 3:**
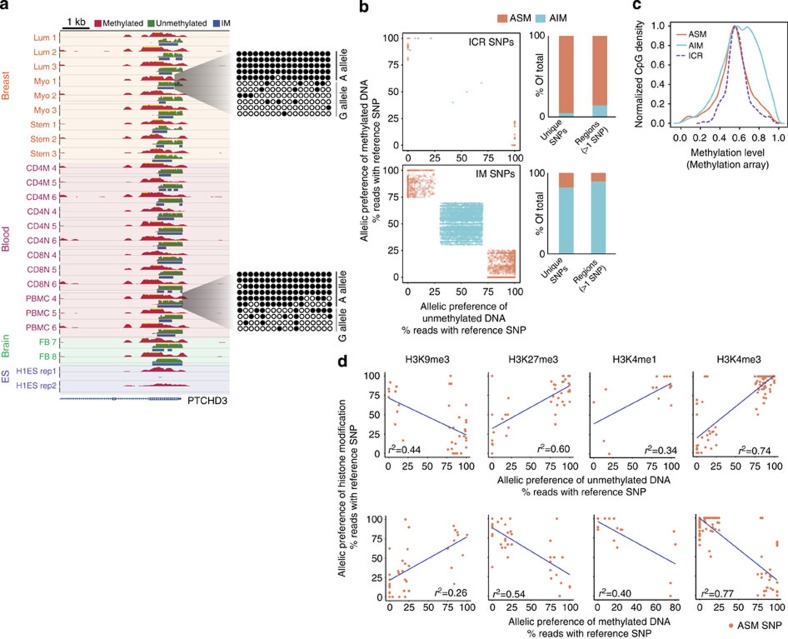
Characterization of ASM and AIM regions. (**a**) A novel ASM region in exon 1 of *PTCHD3* validated by clonal bisulfite sequencing. Height for all tracks shows a signal range from 0 to 50 reads. (**b**) Scatter plots showing separation of ASM and AIM SNPs in imprinting control regions (ICRs) and in all IM regions based on allelic preference in MeDIP-seq and MRE-seq reads. Bar graphs showing relative proportions of ASM and AIM SNPs, and proportions of whole ASM and AIM IM regions based on the presence of >1 ASM or AIM SNP per region. (**c**) Comparison of methylation array levels between CpGs in ICRs, ASM and AIM. A value of 0 is unmethylated, and a value of 1 is fully methylated. (**d**) Comparison of allelic preference between histone modifications, unmethylated DNA (MRE-seq, top panel) and methylated DNA (MeDIP-seq, bottom panel) at ASM SNPs from fetal brain, in which the heterozygous genotype was verified by whole-genome sequencing. A positive correlation indicates that signals are on the same allele, whereas negative correlation indicates that signals are on opposite alleles.

IM CpGs could reflect ASM or allele-independent methylation (AIM). Interestingly, most previously identified ASM has not been connected to allelic gene expression^14^,^17^. To distinguish ASM from AIM, we identified heterozygous single-nucleotide polymorphisms (SNPs) in the autosomal IM regions and determined the allelic preference of sequencing reads from MeDIP/MRE-Seq, where ASM segregated heterozygous SNPs between the two assays (Methods). We identified a total of 2,072 ASM SNPs (721 unique across samples) and 5,895 AIM SNPs (3,262 unique; Fig. 3a,b). We then categorized each IM region as ASM or AIM if it contained two or more ASM or AIM SNPs (Methods). We identified 109 ASM regions and 927 AIM regions (Fig. 3b). From these data, we infer that the methylation pattern underlying a majority of IM regions is allele independent rather than allele specific.

ASM should have approximately 50% methylation at individual CpGs, whereas IM due to intercellular heterogeneity could theoretically fall at any IM value. On the methylation arrays, methylation scores of CpGs within ICRs and our predicted ASM had a relatively narrow distribution centred near 50%. Methylation scores at AIM regions peaked near 50% but had a broader distribution, consistent with the presence of more intercellular variation at individual CpGs, and potentially across neighbouring CpGs (Fig. 3c).

We selected 36 high confidence IM regions (based on MeDIP/MRE-Seq signal strength) for validation by bisulfite, PCR, cloning and sequencing. A total of 35 loci validated as IM (97%), including 12 of 14 ASM (86%; 2 predicted as ASM were found to be AIM; Fig. 3a) and 12 of 12 AIM (100%) regions. Interestingly, the AIM regions exhibited an interspersed mixture of methylated and unmethylated CpGs on each clone (Supplementary Fig. 6 and Supplementary Data 2). Thus, the intermediate signals in H3K4me1, H3K4me3 and DHSs at IM regions cannot be attributed to fully unmethylated alleles in an ASM configuration. Potential configurations include intercellular heterogeneity, differences in neighbouring nucleosomes and differences between individual subunits within each nucleosome.

To examine the relationship between ASM and histone modifications, we measured allelic preference for histone signals at ASM loci and compared this to the allelic preference of MeDIP/MRE-Seq signals at the same locations (Fig. 3d). We focused on heterozygous SNPs from the two fetal brain samples with genotype validated by whole-genome sequencing. At loci classified as ASM, the active chromatin marks H3K4me3 and H3K4me1 showed a clear preference for the unmethylated allele, whereas the repressive mark H3K9me3 had a bias towards the methylated allele. Interestingly, the repressive mark H3K27me3 preferentially occurred on the unmethylated allele. In contrast, histone modifications at AIM and fully unmethylated regions did not exhibit a strong allelic preference (Supplementary Fig. 4).

### IM state is evolutionarily conserved

**Figure 4:**
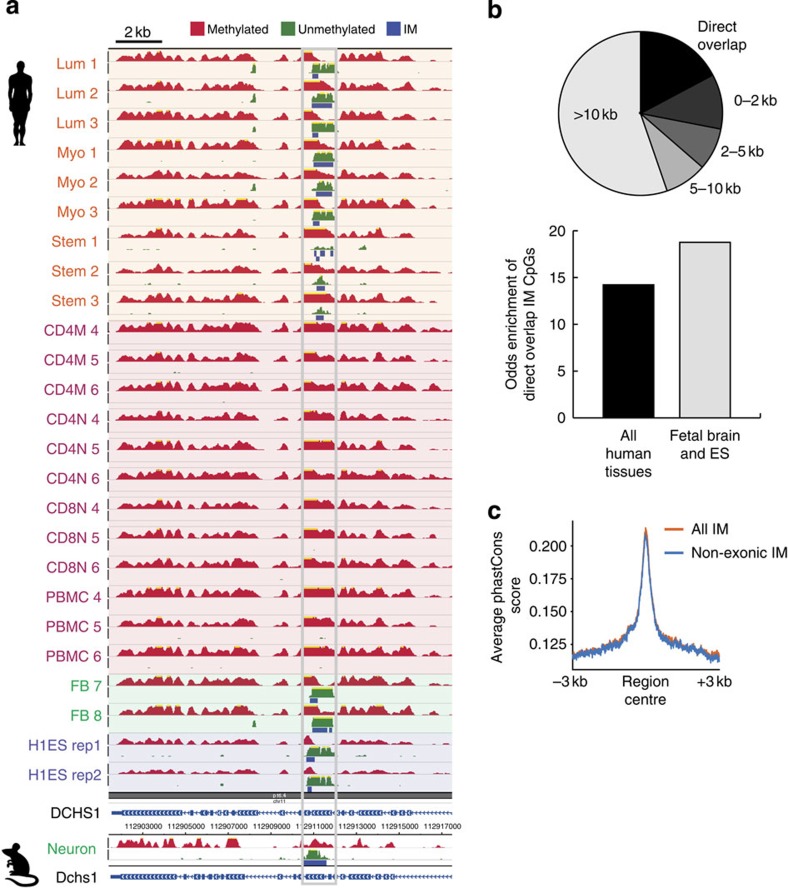
The IM state is conserved in syntenic loci in mouse. (**a**) A novel, tissue-specific human IM region in an internal exon of *DCHS1* shows conserved IM state at the orthologous exon in mouse. Height for all tracks shows a signal range of 0–50 reads. (**b**) The pie chart indicates the distance to the nearest human IM region from each aligned mouse IM region. The bar graph shows the fold-enrichment of overlap between human and mouse IM regions at the CpG level using the complete set of human IM regions and a set restricted to cell types analogous to those in the mouse IM analysis. (**c**) Average phastCons conservation scores over all IM regions and regions that do not overlap coding exons. Scores are based on alignment of 46 vertebrate species.

As a complementary comparative epigenomics approach to investigate whether IM regions may have function, we identified IM states using MeDIP/MRE-Seq data from murine embryonic stem (ES) cells and fetal neurons, taking the union of regions in both cell types as the reference IM set for mouse. A total of 13,623 IM regions were detected in the two mouse samples. We then determined how frequently IM states in the mouse genome were conserved as IM in human. After mapping mouse IM regions to their syntenic loci in the human genome, 17.4% directly overlapped human IM regions, representing a 14-fold enrichment over random expectation (*P*<0.001, *χ*^2^), and suggesting significant cross-species conservation of the IM state (Fig. 4a,b). When restricting the human IM set to only the two human tissues (ES and fetal brain) that were also profiled from mouse, the enrichment increased to nearly 19-fold, suggesting that tissue specificity of IM is also conserved. Averaged phastCons scores over IM regions indicated DNA sequence conservation that reached its maximum at the centre of the region, even when coding exons were excluded (Fig. 4c).

## Discussion

We have defined 18,452 discrete regions of the genome that maintain DNA methylation levels near 50% in one or more cell types across multiple individuals, and are associated with intermediately active rather than suppressed gene expression. Although the precise function of intermediate DNA methylation states is challenging to prove using current methods, the IM signature enriches for regions with multiple indicators of regulatory function, particularly those associated with enhancers. The intermediate level of active histone modification and chromatin accessibility at IM regions implies these are regulatory sites distinct from repressive, fully methylated states or permissive, unmethylated states. Intermediate signals cannot be explained trivially as cell-type differences within tissues, because they are equally intermediate in tissue, unsorted peripheral blood and six highly purified cell types. The strong association of exonic IM with an intermediate level of exon inclusion provides independent validation of the general concept that IM is an epigenomic signature of context-dependent function. Significant interspecies conservation, and conservation among different individuals at IM regions further suggests an important function and potentially a shared mechanism for their establishment and maintenance.

Although an individual CpG is either methylated or unmethylated, our results raise the possibility that local clusters of CpGs provide quantitative instructions for gene regulation or exon usage that exists stably between fully ‘on’ and ‘off’ states within single-cell types. Intriguingly, we approximate that 18% or fewer IM regions are allele-specifically methylated, whereas the majority is allele independent, suggesting that the predominant mode of IM establishment relies on epigenetic heterogeneity at a precise loci within cell populations. Methods to create and abolish IM states in a locus-specific manner will be required to further examine these possibilities^31^.

Widespread intercellular and allelic stochasticity of gene expression has been observed in single-cell mRNA sequencing data^32^,^33^,^34^; however, it is not clear why some genes exhibit stochastic expression while others are uniformly expressed, or if a biological mechanism might be needed to establish or control intercellular differences. A quantitative model of epigenetic gene regulation that includes stable, tissue-specific intermediate states may help explain variations in gene activity between cells of the same type. Although this remains to be proven, it would have far-reaching implications for the multitude of diseases linked to disruption of DNA methylation, including the epigenetic heterogeneity in cancer cells^35^,^36^.

## Methods

### Sample preparation

All assays were performed as part of the repository of the NIH Roadmap Epigenomics Mapping Centers for the reference Human Epigenome Atlas^37^.

### Blood

Buffy coats were obtained from the Stanford Blood Center (Palo Alto, California, USA). Blood was drawn and processed on the same day. Peripheral blood mononuclear cells (PBMCs) were isolated by Histopaque-1077 (Sigma-Aldrich) density-gradient centrifugation according to the manufacturer’s protocol. Further purification of memory CD4^+^, naive CD4^+^ and naive CD8^+^ T lymphocytes was performed using a RoboSep instrument and the isolation kit for each subpopulation (EasySep Human Memory CD4^+^ T Cell Enrichment kit, EasySep Human Naive CD4^+^ T Cell Enrichment kit and Custom Human Naive CD8^+^T Cell Enrichment kit; STEMCELL Technologies). Total PBMCs were karyotyped (Molecular Diagnostic Services) and analysed to determine the percentage of cells in each cell cycle stage (G0/G1, S and G2/M). PBMC and T-cell subpopulations were stained with antibodies (anti-CD3 TRI-COLOR (Invitrogen), anti-CD4 PE (BD Biosciences), anti-CD8 FITC (BD Biosciences), anti-CD4 TRI-COLOR (Invitrogen), anti-CD45RO PE (Invitrogen), anti-CD45RA FITC (BD Biosciences) and anti-CD8 TRI-COLOR (Invitrogen)) and analysed by fluorescence-activated cell sorting for purity. Cells were aliquoted for isolation of either DNA or RNA and were washed in PBS. Cell pellets for RNA purification were resuspended in 1 ml of TRIzol reagent (Invitrogen) and frozen at −80 °C. Cell pellets for DNA purification were flash frozen in liquid nitrogen and stored at −80 °C. Antibodies used included TRI-COLOR-conjugated antibody to CD3 (Invitrogen, MHCD0306), Phycoerythrin-conjugated antibody to CD4 (BD Biosciences, 340419), fluorescein isothiocyanate (FITC)–conjugated antibody to CD8 (BD Biosciences, 561947), TRI-COLOR-conjugated antibody to CD4 (Invitrogen, MHCD0406), PE-conjugated antibody to CD45RO (Invitrogen, MHCD45RO04), FITC–conjugated antibody to CD45RA (BD Biosciences, MHCD45RA01) and TRI-COLOR-conjugated antibody to CD8 (Invitrogen, MHCD0806). All antibodies were used according to the manufacturer’s instructions.

### Breast

Breast tissues were obtained from disease-free premenopausal women undergoing reduction mammoplasty in accordance with institutional review board protocol 10-01563 (previously CHR 8759-34462-01). All tissues were obtained as de-identified samples and were linked only with a minimal data set (age, ancestry and, in some cases, parity/gravidity). All study subjects provided written informed consent. Tissue was dissociated mechanically and enzymatically^38^. Briefly, tissue was minced and dissociated in RPMI 1640 with L-glutamine and 25 mm HEPES (Fisher) supplemented with 10% fetal bovine serum (FBS; JR Scientific), 100 U ml^−1^ penicillin, 100 μg ml^−1^ streptomycin sulfate, 0.25 μg ml^−1^ Fungizone, 50 μg ml^−1^ gentamicin sulfate (Lonza), 200 U ml^−1^ collagenase 2 (Worthington) and 100 U ml^−1^ hyaluronidase (Sigma-Aldrich) at 37 °C for 16 h. The cell suspension was centrifuged at 395 *g* for 10 min, and the pellet was washed with RPMI 1640 supplemented with 10% FBS. Clusters enriched in epithelial cells (referred to as organoids) were recovered after serial filtration through 150-μm nylon mesh (Fisher) and 40-μm nylon mesh (Fisher). The final filtrate contained primarily mammary stromal cells (fibroblasts, immune cells and endothelial cells) and some single epithelial cells. After centrifugation at 290 *g* for 5 min, the epithelial organoids and filtrate were frozen for long-term storage. The day of cell sorting, epithelial organoids were thawed and further digested with 0.5 g l^−1^ trypsin, 0.5 mM EDTA, pH 8.0, and dispase DNase I (STEMCELL Technologies). Generation of single-cell suspensions was monitored visually. Single-cell suspensions were filtered through a 40-μm cell strainer (Fisher), spun down and allowed to regenerate in mammary epithelial cell growth medium (Lonza) supplemented with 2% FCS for 60–90 min at 37 °C. This regeneration step enables quenching of trypsin and re-expression of the cell surface markers before staining, which was need because their extracellular domains had been cleaved by trypsin.

The single-cell suspension obtained was stained for cell sorting with three human-specific primary antibodies, including antibody to CD10 labelled with PE-Cy5 (BD Biosciences, 555376) to isolate myoepithelial cells, antibody to CD227/MUC1 labelled with FITC (BD Biosciences, 559774) to isolate luminal epithelial cells and antibody to CD73 labelled with PE (BD Biosciences, 550257) to isolate a stem cell-enriched cell population, as well as with biotinylated antibodies for lineage markers, including antibodies to CD2 (555325), CD3 (555338), CD16 (555405), CD64 (555526) (all from BD Biosciences), CD31 (Invitrogen, MHCD3115), CD45 (BioLegend, 304003) and CD140b (BioLegend, 323604) to specifically remove haematopoietic (CD16- and CD64-positive), endothelial (CD31-positive), leukocytic (CD2-, CD3- or CD45-positive) and mesenchymal (CD140b-positive) lineage cells by negative selection. Sequential incubation with primary antibodies was performed for 20 min at room temperature in PBS with 1% BSA, and cells were washed in PBS with 1% BSA. Biotinylated primary antibodies were detected with a secondary antibody to human immunoglobulin labelled with streptavidin–Pacific Blue conjugate (Invitrogen, S11222). After incubation, cells were washed once in PBS with 1% BSA, and cell sorting was performed using a FACSAria II cell sorter (BD Biosciences).

### Fetal brain

Post-mortem human fetal neural tissues were obtained from a case of twin non-syndrome fetuses whose death was attributed to environmental/placental aetiology. Tissues were obtained with appropriate consent according to the Partner's Healthcare/Brigham and Women's Hospital Institutional Review Board guidelines (protocol 2010P001144). All samples and tissues were de-identified and linked only with a minimal data set (age, sex and brain location). Fetal brain tissue and fetal neural progenitor cells were derived from manually dissected regions of the brain (telencephalon), specifically the neocortex (pallium; GSM669614 and GSM669615). Tissues were minced and dissociated by mechanical agitation (gentleMACS device) during enzymatic treatment with papain according to the manufacturer’s protocol (Miltenyi Biotec, Neural Tissue Dissociation kit). Cell suspensions were then washed twice in Dulbecco’s modified Eagle medium (DMEM) and plated at low density in human NeuroCult NS-A medium (STEMCELL Technologies) supplemented with heparin, epidermal growth factor (20 ng ml^−1^) and fibroblast growth factor (10 ng ml^−1^) in ultra-low-attachment cell culture flasks (Corning).

### H1 embryonic stem cells

H1 cells were grown in mTeSR1 medium on Matrigel (BD Biosciences) for ten passages on 10 cm^2^ plates and harvested at passage 27. Cells were harvested by scraping before snap freezing for DNA isolation. Cells were also harvested from passages 30 and 32 and divided for isolation of DNA, RNA and chromatin^23^.

### Mouse embryonic stem cells and fetal neurons

Mouse embryonic stem cells (E14) were plated onto gelatin-coated dishes in DMEM (GIBCO), supplemented with 15% heat-inactivated FBS (GIBCO), 0.055 mM mercaptoethanol (GIBCO), 2 mM L-glutamine, 0.1 mM MEM non-essential amino acid, 5,000 U ml^−1^ penicillin/streptomycin and 1,000 U ml^−1^ of LIF (Millipore ESG1107) in an incubator set at 37 °C and 5% CO_2_ (MeDIP-Seq: GSM881346; MRE-Seq: GSM881347). Studies were conducted with the approval of the University of California San Diego Internal Review Board and Animal Studies Committee.

Cortical neurons from E15 CD-1 mice (Charles River Laboratories) were processed as described for mouse dorsal root ganglion primary cultures (protocol number 20140044)^39^. The animal studies committee at the Washington University School of Medicine approved the experiments. Gestational day 12.5–13 embryos were removed and cells were dissected out and dissociated using 0.5 ml trypsin/EDTA (0.05%/0.02%; Tissue Culture Support Center, Washington University, St Louis, MO, USA) for no longer than 15 min at 37 °C with two or three times of gentle shaking by hand. The reaction was stopped by addition of 0.5 ml of DMEM/10% FBS. All subsequent procedures were performed in a laminar flow hood under sterile condition. Cells were gently triturated by pipetting them up and down with a sterile P1000 pipetman less than ten times. No cell clumps should be visible after this trituration. Cell suspensions were centrifuged at 2,000 r.p.m. for 5 min at room temperature. Supernatants were completely removed. Cell pellets were washed twice with 1 ml of DMEM/10% FBS. Cells were resuspended in either DMEM/10% FBS or growth medium, plated and cultured in neurobasal medium (Invitrogen #21103-049) with B27 serum-free supplement (Invitrogen #17504-044), glutamine (5 mM) and penicillin/streptomycin. Cells were typically seeded at a density of ‘one forebrain per 24-well plate’. Neurons were stimulated with KCl (55 mM) at 5 days in vitro for 4 h (ref. 40).

### Chromatin immunoprecipitation sequencing

Standard operating procedures for ChIP-seq library construction are available at http://www.roadmapepigenomics.org/protocols/type/experimental/. ChIP-seq library construction involves the following protocols in order: (i) cross-linking of frozen cell pellet, (ii) DNA sonication using Sonic Dismembrator 550 and (iii) SLX-PET protocol for Illumina sample preparation. Antibodies used in this study were subjected to rigorous quality assessment to meet Reference Epigenome Mapping Quality Standards (http://www.roadmapepigenomics.org/protocols) including western blotting of whole-cell extracts, 384 peptide dot blot (Active Motif MODified Histone Peptide Array) and ChIP-seq using control cell pellets (HL60). Antibody vendor, catalogue number and lot are provided along with ChIP-seq library construction details as part of the metadata associated with all ChIP-seq data sets and are available through GEO and the NCBI epigenomics portals (for example, http://www-ncbi-nlm-nih-gov.beckerproxy.wustl.edu/geo/query/acc.cgi?acc= GSM613811). Final library distributions were calculated using an Agilent Bioanalyzer and quantified by fluorometric quantification (Qubit, Life Technologies). Libraries were sequenced using single-end 76 nt sequencing chemistry on an Illumina GAiix or HiSeq2000 following the manufacturer’s protocols (Illumina) as either single or multiplexed libraries using custom index adapters added during library construction.

Sequencing reads were aligned to NCBI GRCh37-lite reference using Burrows-Wheeler Alignment tool (BWA) 0.6.2-r126 with default parameters. MethylQA (an unpublished C programme; available at http://methylqa.sourceforge.net/) was used to directionally extend aligned reads to the average insert size of DNA fragments (150 bp) and to generate a bigWig file for downstream visualization. Reads with BWA mapping quality scores ≪10 were discarded and reads that aligned to the same genomic coordinate were counted only once.

### Methylation-sensitive restriction enzyme-seq

Methylation-sensitive restriction enzyme (MRE)-seq was performed as in Maunakea *et al*.^22^, with modifications as detailed below. Five parallel restriction enzyme digestions (*Hpa*II, *Bsh*1236I, *Ssi*I(AciI) and *Hin*6I (Fermentas), and *Hpy*CH4IV (NEB)) were performed, each using 1 μg of DNA per digest for each of the skin cell type samples. Five units of enzyme were initially incubated with DNA for 3 h and then an additional five units of enzyme was added to the digestion for a total of 6 h of digestion time. DNA was purified by phenol/chloroform/isoamyl alcohol extraction, followed by chloroform extraction using phase lock gels. Digested DNA from the different reactions was combined and precipitated with one-tenith volume of 3 M sodium acetate (pH 5.2) and 2.5 volumes of ethanol. The purified DNA was size selected and purified (50–300 bp) by gel electrophoresis and Qiagen MinElute extraction. Library construction was performed as per the Illumina Genomic DNA Sample Prep Kit protocol with the following modifications. During the end-repair reaction, T4 DNA polymerase and T4 PNK were excluded and 1 μl of 1:5 diluted Klenow DNA polymerase was used. For the adapter ligation reaction, 1 μl of 1:10 diluted PE adapter oligo mix was used. Ten microlitres from the 30 μl of purified adapter ligated DNA was used for the PCR enrichment reaction with PCR PE Primers 1.0 and 2.0. PCR products were size selected and purified (170–420 bp) by gel electrophoresis and Qiagen Qiaquick extraction. DNA libraries were checked for quality by Nanodrop (Thermo Scientific) and Agilent DNA Bioanalyzer (Agilent).

Reads were aligned to hg19 using the BWA and pre-processed using methylQA (an unpublished C programme; available at http://methylqa.sourceforge.net/). MRE reads were normalized to account for differing enzyme efficiencies and methylation values were determined by counting reads with CpGs at fragment ends^22^. To enable comparison between MRE-seq data from blood, brain and breast samples that used three restriction enzymes and skin cell types that used five restriction enzymes, skin cell-type MRE reads that resulted from the use of additional restriction enzymes (*Bsh*1236I and *Hpy*CH4IV) were removed. Detailed library construction protocols for MRE-seq, methylated DNA immunoprecipitation-seq (MeDIP-seq), ChIP-seq, RNA-seq and miRNA-seq are publicly available at the NIH Roadmap Epigenomics project website http://www.roadmapepigenomics.org/protocols/type/experimental/.

### Methylated DNA immunoprecipitation-seq

MeDIP-seq was performed as in Maunakea *et al*.^22^ Five micrograms of genomic DNA were sonicated to a fragment size of ~100–400 bp using a Bioruptor sonicator (Diagenode). End-repair, addition of 3′-A bases and PE adapter ligation with 2 μg of sonicated DNA was performed as per the Illumina Genomic DNA Sample Prep Kit protocol. Adapter-ligated DNA fragments were size selected to 166–366 bp and purified by gel electrophoresis. DNA was heat denatured and then immunoprecipitated with 5-methylcytidine antibody (Eurogentec; 1 μg of antibody per 1 μg of DNA) in 500 μl of immunoprecipitation buffer (10 μM sodium phosphate, pH 7.0, 140 mM sodium chloride and 0.05% Triton X-100) overnight at 4 °C. Antibody/DNA complexes were isolated by addition of 1 μl of rabbit anti-mouse IgG secondary antibody (2.4 mg ml^−1^, Jackson Immunoresearch) and 100 μl protein A/G agarose beads (Pierce Biotechnology) for 2 h at 4 °C. Beads were washed six times with immunoprecipitation buffer and then DNA was eluted in TE buffer with 0.25% SDS and 0.25 mg ml^−1^ of proteinase K for 2 h at 50 °C. DNA was then purified with the Qiagen Qiaquick kit and eluted in 30 μl EB buffer. Ten microlitres of DNA were used for a PCR-enrichment reaction with PCR PE Primers 1.0 and 2.0. PCR products were size selected (220–420 bp) and purified by gel electrophoresis. Methylated DNA enrichment was confirmed by PCR on known methylated (SNRPN and MAGEA1 promoters) and unmethylated (a CpG-less sequence on chromosome 15 and glyceraldehyde 3-phosphate dehydrogenase promoter) sequences. DNA libraries were checked for quality by Nanodrop (Thermo Scientific) and Agilent DNA Bioanalyzer (Agilent). Reads were aligned to hg19 using BWA and pre-processed using methlyQA.

### mRNA-Seq

Standard operating procedures for RNA-Seq library construction are available at (http://www.roadmapepigenomics.org/protocols/type/experimental/) or by request. RNA-Seq library construction involves the following SOPs in order: (i) Purification of polyA+ mRNA and mRNA(−) Flow-Through Total RNA using MultiMACS 96 Separation Unit; (ii) Strand Specific 96-Well cDNA Synthesis; (iii) Strand Specific 96-well Library Construction for Illumina Sequencing. Briefly, polyA+ RNA was purified using the MACS mRNA isolation kit (Miltenyi Biotec), from 2 to 10 μg of total RNA with a RNA integrity number ≥7 (Agilent Bioanalyzer) as per the manufacturer’s instructions. The process included on-column DNaseI treatment (Invitrogen). Double-stranded cDNA was synthesized from the purified polyA+ RNA using the Superscript II Double-Stranded cDNA Synthesis kit (Invitrogen) and 200 ng random hexamers (Invitrogen). After first strand synthesis, dNTPs were removed using two volumes of AMPure XP beads (Beckman Genomics). GeneAmp (Invitrogen) 12.5 mM dNTPs blend (2.5 mM dCTP, 2.5 mM dGTP, 2.5 mM dATP, 5.0 mM dUTP) was used in the second strand synthesis mixture in the presence of 2 μg of ActinomycinD. Double-stranded cDNA was purified using two volumes of Ampure XP beads, fragmented using Covaris E series shearing (20% duty cycle, Intensity 5, 55 s), and used for paired-end sequencing library preparation (Illumina). Before library amplification uridine digestion was performed at 37 °C for 30 min following with 10 min at 95 °C in Qiagen Elution buffer (10 mM Tris-Cl, pH 8.5) with 5 U of Uracil-N-Glycosylase (AmpErase). The resulting single-stranded sequencing library was amplified by PCR (10–13 cycles) to add Illumina P5 and P7 sequences for cluster generation. PCR products were purified on Qiaquick MinElute columns (Qiagen), and assessed and quantified using an Agilent DNA 1000 series II assay and Qubit fluorometer (Invitrogen), respectively. Libraries were sequenced using paired-end 76 nt sequencing chemistry on a cBot and Illumina GA_iix_ or HiSeq2000 following the manufacturer’s protocols (Illumina).

### Whole-genome sequencing

Two micrograms of extracted DNA were sheared for 55 s using a Covaris E210 focused ultra-sonicator (Covaris Inc.) at 20% Duty cycle, 5% Intensity and 200 Cycles per burst. The sheared products were separated on an 8% Novex TBE gel (Invitrogen Canada, Inc.) and the 300–500 bp size fraction was excised and eluted into 300 μl of elution buffer containing 5:1 (vol/vol) LoTe (3 mM Tris-HCl, pH 7.5, 0.2 mN EDTA)/7.5 M ammonium acetate. The elute was purified from the gel slurry by centrifugation through a Spin-X centrifuge tube filter (Fisher Scientific Ltd) and EtOH precipitated. A paired-end library was constructed from the purified DNA following Illumina’s protocol (Illumina Inc.). Briefly, the DNA was subjected to end-repair and phosphorylation by T4 DNA polymerase, Klenow DNA Polymerase and T4 Polynucleotide Kinase, respectively, in a single reaction, then 3′ A overhangs were generated by Klenow fragment (3′ to 5′ exo minus). The modified DNA fragments were ligated to Illumina PE adapters, and the adapter-ligated products purified through a QIAquick spin column (Qiagen Inc.). The ligation products were subjected to six to ten cycles of PCR amplification using Illumina’s PE primer set and Phusion DNA Polymerase (Thermo Fisher Scientific Inc.). The amplified products were separated from non-amplifiable damaged DNA fragments and adapter products on an 8% Novex TBE gel (Invitrogen Canada, Inc.) and purified as above. The purified Illumina library was assessed using an Agilent DNA 1000 Series II assay (Agilent Technologies Canada Inc.), quantified by qPCR according to the KAPA Biosystems Library Quantification Kit protocol (D-Mark Biosciences) and sequenced on Illumina’s HiSeq2000 machines using paired-end 101 nt chemistry.

### Illumina 450K Infinium array

Bisulfite conversion was performed on 1 μg of genomic DNA using the EZ DNA methylation kit (Zymo Research) as per the manufacturer’s alternative incubation conditions protocol. The bisulfite-converted DNA was amplified and hybridized to an Infinium HumanMethylation450 beadchip (Illumina) following the Infinium HD methylation assay protocol at the UCSF Genomics Core facility. Methylation levels (beta values) were determined using the Methylation Module of the Illumina GenomeStudio software.

### Bisulfite treatment and library construction for WGBS

One to five micrograms of gDNA were sonicated to an approximate size range of 200–400 bp. Size selection was achieved by PAGE gel and yielded DNA fragments of 200–300 bp. DNA was quantified by fluorescent incorporation (Qubit, Invitrogen). The library preparation included end-repair, an addition with NEBNext DNA library prep reagent set for Illumina (NEB) or Illumina Sample Prep Kit reagents. Methylated adaptors were ligated and size selection (325–525 bp) was performed to remove excess free adaptors. The ligated DNA was quantified by Qubit, and approximately 100 ng DNA was used for bisulfite conversion. Methylated-adaptor ligated to unmethylated lambda-phage DNA (Promega) was used as an internal control for assessing the rate of bisulfite conversion. The ratio of target library to Lambda was 1,600:1. Bisulfite conversion of the methylated adapter-ligated DNA fragments followed the Formalin-fixed, Paraffin-embedded Tissue Samples Protocol from Qiagen’s Epitect Bisulfite Kit. Cleanup of the bisulfite-converted DNA was performed, and a second round of conversion was applied. Enrichment of adaptor-ligated DNA fragments was accomplished by dividing the template into five aliquots followed by eight cycles of PCR with Illumina PE PCR primers. Post PCR size-selection of the PCR products from the five reactions was achieved by PAGE gel. Following 100 bp paired-end sequencing on a HiSeq2000, sequence reads were aligned and processed through the Bismark pipeline.

### Targeted bisulfite PCR and sequencing

Further validation of genome-wide data, particularly sites with apparent intermediate DNA methylation, was performed by bisulfite sequencing. Total genomic DNA underwent bisulfite conversion following established protocol^41^ with a modified conversion conditions of: 95 °C for 1 min, 50 °C for 59 min for a total of 16 cycles. The regions of interest were amplified with bisulfite PCR primers and were subsequently cloned using pCR2.1/TOPO (Invitrogen). Single colony PCR and sequencing (QuintaraBio) provided contigs that were aligned for analysis.

### Genomic features

CpG islands, untranslated regions (UTRs), gene bodies, exons and introns were RefSeq gene annotations downloaded from the UCSC Genome Browser (hg19). Promoters were defined as the 3-kb upstream of the transcription start site (TSS) for all RefSeq genes. Intergenic regions were defined as all regions outside RefSeq gene bodies and promoters.

### IM detection algorithm

We developed a maximum scoring segment algorithm to identify regions of overlapping MeDIP-Seq and MRE-Seq signals (Supplementary Fig. 1). For each sample, total MeDIP-Seq read counts were normalized to 50 M, and MRE-Seq read counts were normalized to 35 M (ref. 20). Given normalized MeDIP-Seq and MRE-Seq read densities across all CpGs, the algorithm traces through each CpG sequentially, comparing read counts from both assays. An arbitrary score proportional to the read density was increased when the signals overlapped and decreased when they did not, and an additional penalty proportional to the distance between CpGs was assigned. When the score returned to zero at some distance following the initialization of an IM region, the end point of the region was defined as the position with the highest score following the start site.

The IM detection algorithm and related data sets can be downloaded at http://epigenome.wustl.edu/Intermediate_Methylation/.

### IM data filters

To limit the false detection of IM, we determined a length and score threshold by comparing IM calls derived from our data to calls from randomly shuffled data. Paired MeDIP-Seq and MRE-Seq read counts were randomly reassigned to CpGs, maintaining their paired relationship. The length and score distributions of IM calls from 1,000 random simulations were then compared with actual distributions, and cutoffs were selected to achieve an estimated false-positive rate of less than 1%. The minimum score was therefore set to 8.0, and the minimum length set to 100 bp (length cutoff was applied to the reference IM set).

### Reference IM list assembly

The reference list of IM regions is intended as a summary of the IM landscape across all available samples, and represents the union of all IM regions that passed the data filters. IM regions within 100 bp were merged across all samples. We additionally required each reference list IM region to be present in at least 2 of the 25 samples analysed to further reduce false positives. These tasks were performed using applications from the BEDtools suite^42^. The reference set and sample-specific IM regions can be found at http://epigenome.wustl.edu/Intermediate_Methylation/.

### Hierarchical clustering

To compare methylation at IM regions across all samples, we clustered samples based on three separate metrics: MeDIP-Seq read counts (distance metric=Canberra); MRE-Seq read counts (distance metric=Canberra) and binary presence or absence of IM state for each sample (distance metric=Jaccard). We performed hierarchical clustering separately for each metric (clustering method=average).

### DNase I hypersensitivity

The set of DHSs was previously generated for the ENCODE database by combining peak calls from 41 different cell lines^43^.

### VISTA enhancer validation

Human and mouse IM regions were intersected with the complete set of VISTA enhancers. The VISTA Project selects candidate enhancers based on ChIP-Seq and sequence conservation^28^. Regions were tested using *in vivo* reporter assays within mammalian embryos.

### SNP heterozygosity and ASM tests

We analysed only SNPs that fell within reference IM regions, had a minimum read coverage of nine reads in both MeDIP and MRE assays (a maximum read cutoff of 100 was also applied to MRE data to limit influence of PCR bias), and were previously annotated in the database of single-nucleotide polymorphisms (dbSNPs)^44^. We called a SNP heterozygous if each allele was represented in at least 30% of MeDIP or MRE reads. As whole-genome sequencing data are available for one of the twin fetal brain samples, we measured the accuracy of the predicted genotypes for each of the corresponding twin brain samples. At loci where genotype was known from whole-genome sequencing, our criteria for predicting heterozygosity from MeDIP/MRE-Seq achieved a false-positive rate below 3% for each sample.

Of the SNPs categorized as heterozygous, we further classified them as allele-specifically methylated if they met the following criteria: (i) At least 75% of MeDIP-Seq reads contained the same allele identity at the heterozygous locus, whereas at least 75% of MRE reads contained a variant allele; (ii) at least one assay (MeDIP-Seq or MRE-Seq) showed a statistically significant skew towards one allele (*P*<0.01, Fisher’s exact test).

Heterozygous SNPs qualified as allele-independent methylation if they met the following criteria: (i) neither MeDIP-Seq nor MRE-Seq had more than 70% of reads with the same allele identity at the heterozygous SNP locus; (ii) neither assay showed a statistically significant skew towards an allele (*P*>0.01, Fisher’s exact test).

We classified an IM region as ASM if it contained two or more ASM SNPs from any sample and no AIM SNPs. Similarly, we classified an IM region as AIM if it contained two or more AIM SNPs and no ASM SNPs. One hundred and thirty-six regions contained both ASM and AIM SNPs, and were ignored.

### Comparing regions with different methylation levels

To compare ChIP-Seq signal levels between similarly sized and distributed IM, methylated and unmethylated regions, we examined regions that fell within the boundaries of the union of all sample IM regions, which are IM in at least one but not necessarily all samples. In a given sample, a region was defined as unmethylated if it carried four or more MRE-Seq reads and no MeDIP-Seq reads over at least 75% of CpGs within the region. Conversely, a region was defined as methylated if it carried four or more MeDIP-Seq reads and no MRE-Seq reads at 75% of CpGs or greater. These region definitions were used for comparison of histone modifications, DHS levels and expression of nearby genes.

Histone and DHS signals were measured in 100 bp bins starting from the centre of each region and extending ±5 kb. Signal level was calculated as reads per kilobase per millions of reads sequenced (RPKM), and a generalized additive model was used to approximate the shape of the signal within each group of regions (methylated, unmethylated and IM) across all bins within a 95% confidence interval^45^. CD8 Naïve and PBMC ChIP-Seq were taken from donors that were not included in the IM analysis (CD8N H3K4me3: GSM613811; CD8N H3K4me1: GSM613814; PBMC H3K4me3: GSM1127126; PBMC H3K4me1: GSM1127143). DNase-Seq data were from H1ES and fetal brain donors not included in the IM analysis (H1ES: GSM878616, fetal brain: GSM878651).

### Gene expression and relative exon expression

Methylated, unmethylated and IM regions were associated with NCBI Reference Sequence (RefSeq) genes if they fell within 10 kb of the transcription start site of that gene (Total gene-associated regions: IM=6,776; methylated=3,270; unmethylated=5,605). Gene expression levels were measured as RPKM for each annotated gene transcript associated with a region.

Regions were associated with exons if they occurred within 1 kb of the exon (IM exons=14,336; methylated exons=6,642; unmethylated exons=9,331). Relative exon expression was measured using the following formula:




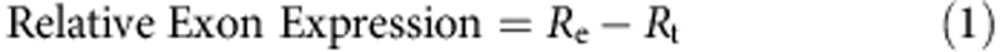




where *R*_e_ is the RPKM value for a given exon and *R*_t_ is the RPKM value for the transcript containing that exon.

### Comparison of human and mouse IM

Mouse coordinates (mm9) were mapped to orthologous human regions using the UCSC LiftOver tool with minMatch parameter set to 0.8 (80% sequence identity). Additional data tables can be downloaded at http://epigenome.wustl.edu/Intermediate_Methylation/.

## Additional information

**Accession codes:** All high-throughput sequencing data have been deposited in the NCBI Gene Expression Omnibus under project accession code GSE16368.

**How to cite this article:** Elliott, G. *et al*. Intermediate DNA methylation is a conserved signature of genome regulation. *Nat. Commun.* 6:6363 doi: 10.1038/ncomms7363 (2015).

## Supplementary Material

Supplementary Figures, Supplementary Methods and Supplementary References Supplementary Figures 1-6, Supplementary Methods and Supplementary References

Supplementary Data 1 Vista enhancer validation of human and mouse IM regions.

Supplementary Data 2 Targeted bisulfite PCR and sequencing validation of select IM regions.
