# Supplementary material for: Intermediate DNA methylation is a conserved signature of genome regulation
Source: Nat Commun. 2015 Feb 18;6:6363. doi: 10.1038/ncomms7363 (PMC4333717; doi:10.1038/ncomms7363)
Supplement: Supplementary Figures, Supplementary Methods and Supplementary References — Supplementary Figures 1-6, Supplementary Methods and Supplementary References [file ncomms7363-s1.pdf]

**Supplementary Information**

**Supplementary Figures**

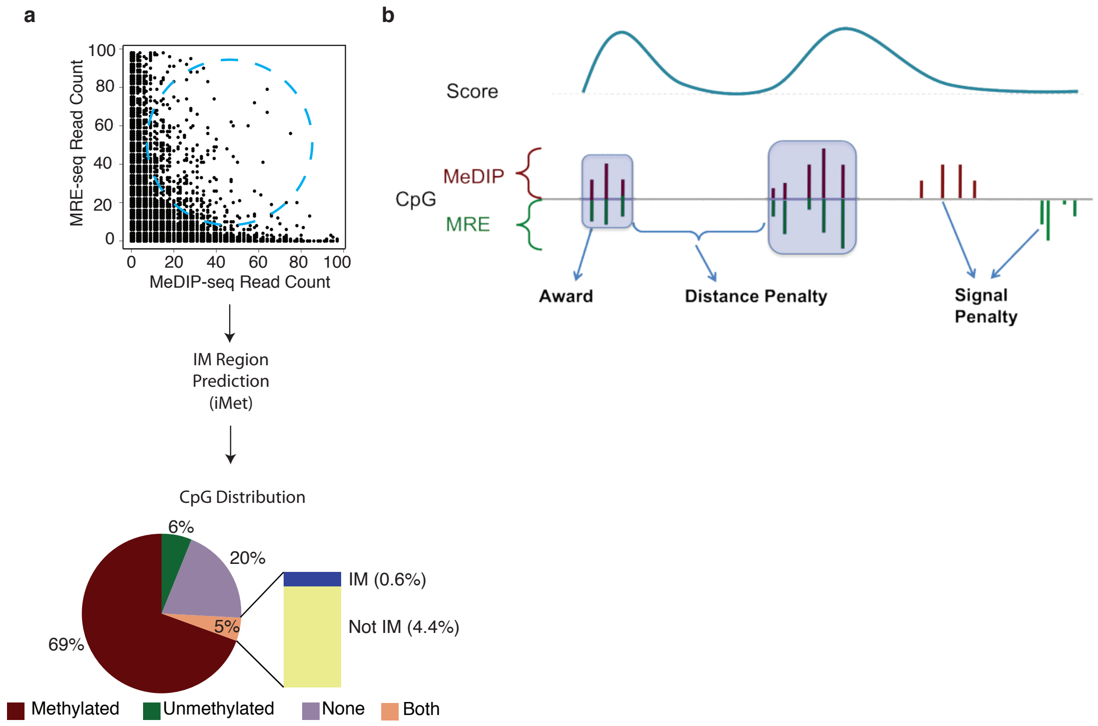


**Supplementary Figure 1. Overview of IM detection**

(a) Conceptual overview of the pipeline for IM detection using a breast luminal epithelial cell sample as an example: scatter plot of MeDIP-Seq and MRE-Seq read counts at 100,000 randomly selected CpGs showing presence of co-occurring signal at a subset of loci (circled). The proportion of CpGs covered by MeDIP-Seq only, MRE-Seq only, or both (>1 read in both assays) are shown in the pie chart. (b) Schematic of the IM detection algorithm. A putative IM region was initiated when a CpG with strong MeDIP-Seq and MRE-Seq signals is detected. The region score was increased when subsequent IM CpGs were encountered, and penalized according to distance between CpGs or when CpGs with non-overlapping MeDIP-Seq/MRE-Seq were encountered. When the score decreased to zero, the point of initiation and the point of the highest score were returned as IM region boundaries.

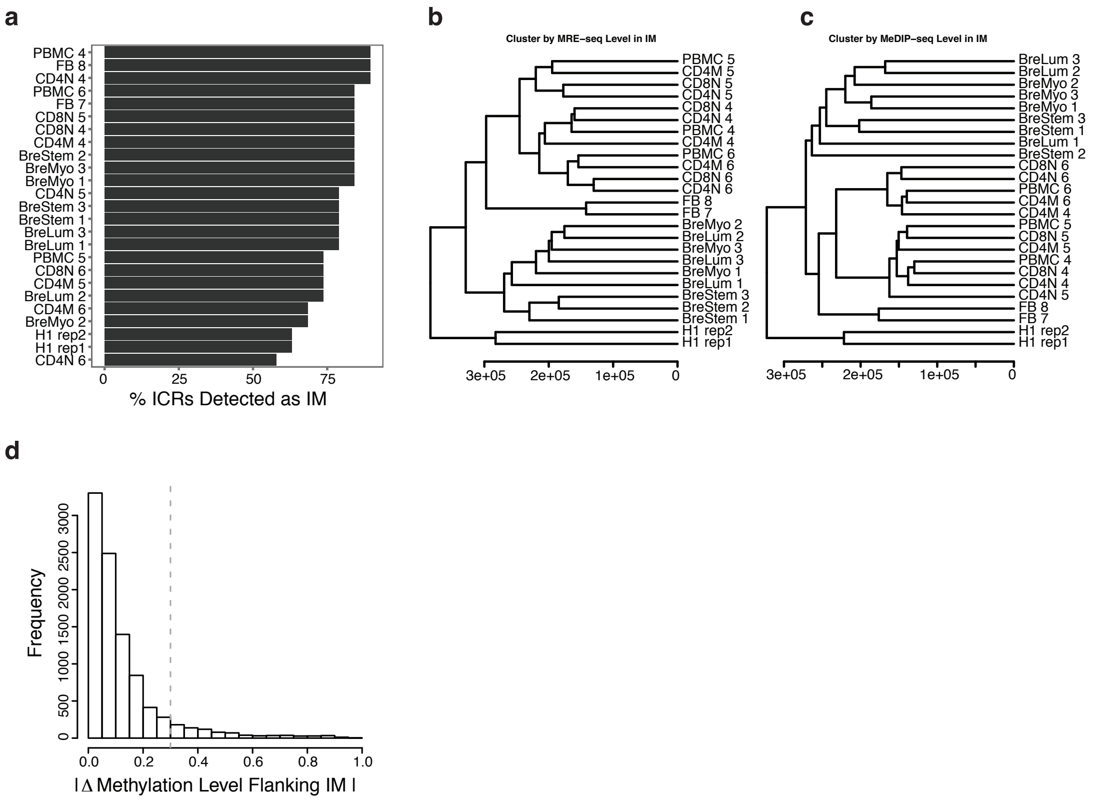


**Supplementary Figure 2. Sensitivity of IM to ICRs and clustering of samples**

(a) Sensitivity of IM detection algorithm to 19 known ICRs. For each sample, the percentage of ICRs that overlap with IM regions are shown. These ICRs have previously been identified as parent-of-origin dependent allele-specific methylation within precise boundaries[1]. (b) Hierarchical clustering of samples based on MRE-Seq read density within IM regions. Distance metric was Canberra; clustering method was average. (c) Clustering of samples based on MeDIP-Seq read density within IM regions. Distance metric was Canberra; clustering method was average. (d) A histogram plot of the difference between WGBS methylation levels of the right and left flanking 100bp regions to each IM region from H1 ES cells. The dotted line marks a methylation difference of 0.3 (or 30%), which is typically the minimum threshold for calling differential methylation. 91% of autosomal IM regions have a methylation difference of less than 0.3 between flanking regions.

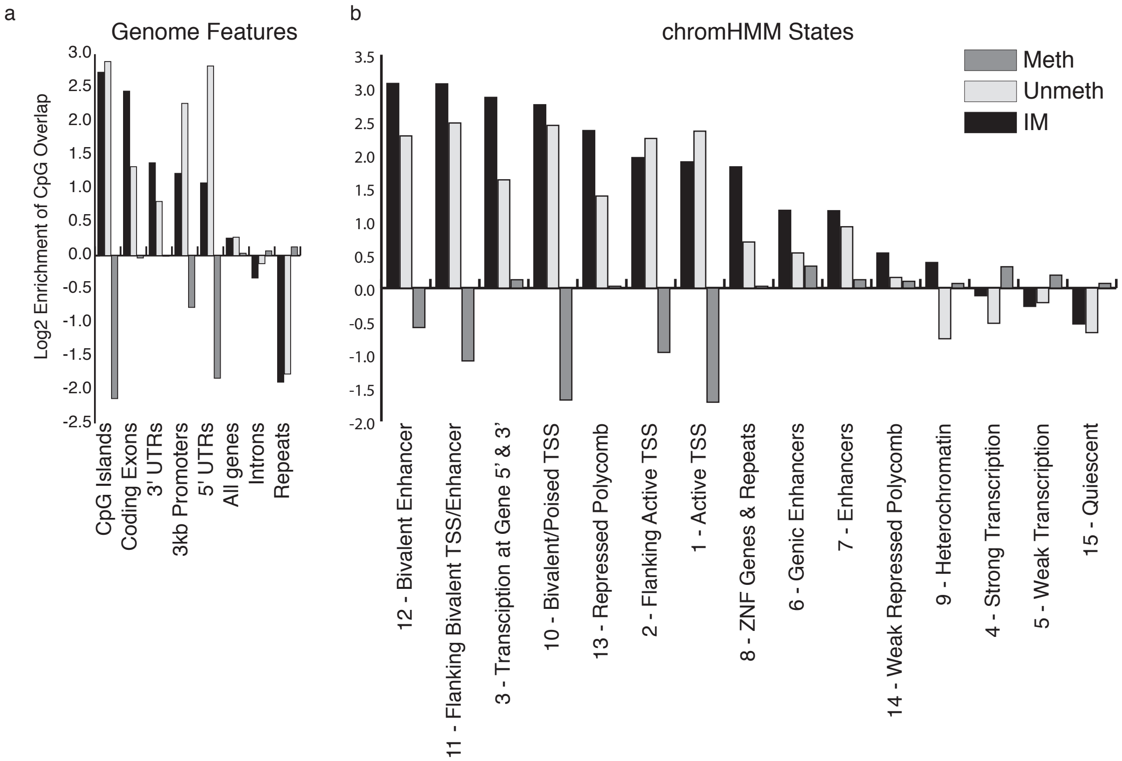


**Supplementary Figure 3. Genomic enrichment of IM, methylated, and unmethylated CpGs**

(a) Log-odds enrichment of unmethylated (MRE-Seq only), methylated (MeDIP-Seq only), and IM CpGs over gene features. (b) Log-odds enrichment of unmethylated, methylated, and IM CpGs over 15 chromHMM functional annotations[2].

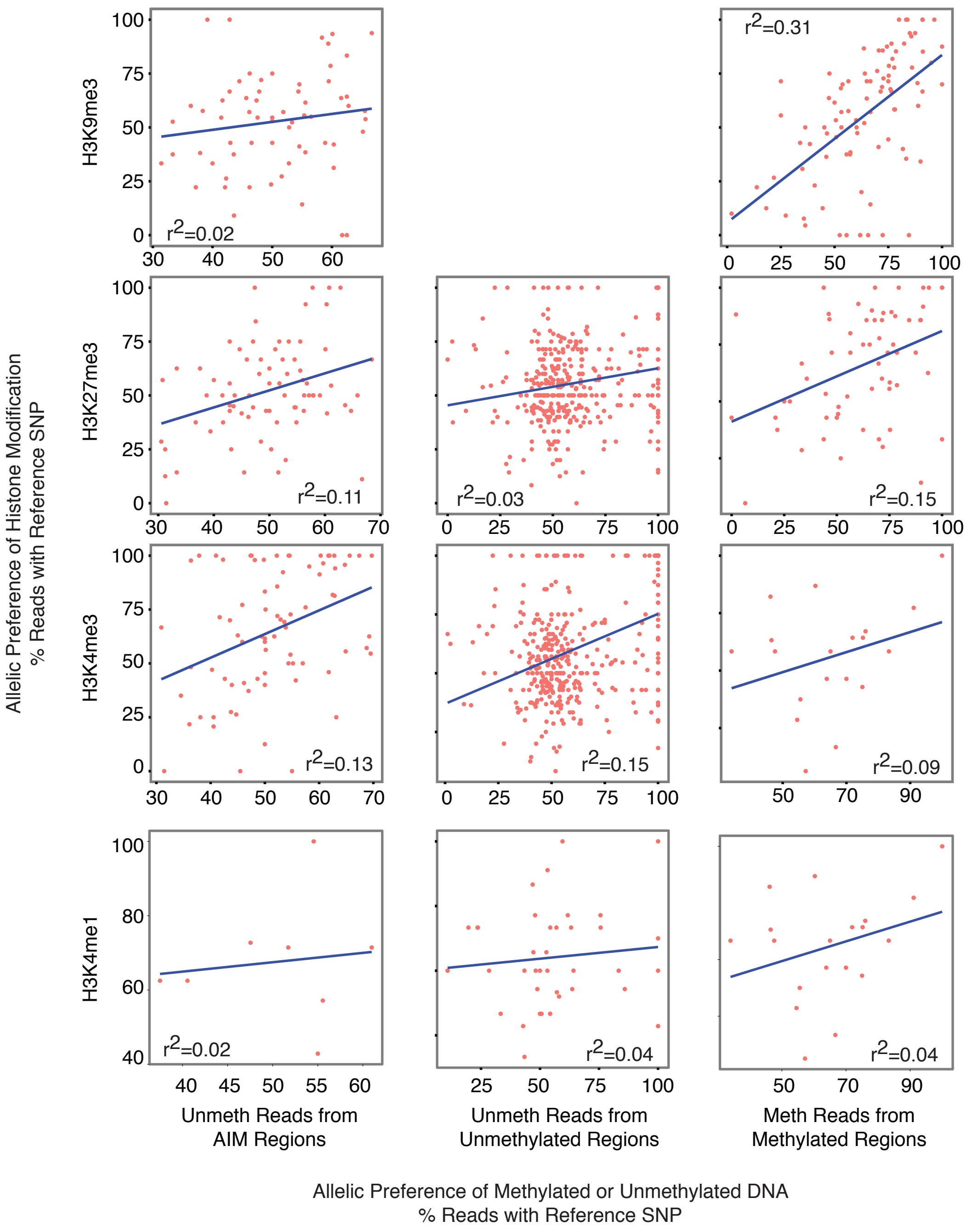


**Supplementary Figure 4. Histone allelic preference at non-ASM regions**

Correlation of allelic preference between histone modifications and MRE-Seq (Unmeth) or MeDIP-Seq (Meth) at loci that are not ASM: AIM, unmethylated (MRE-Seq only), and methylated (MeDIP-Seq only) regions.

Extended Data Fig. 5

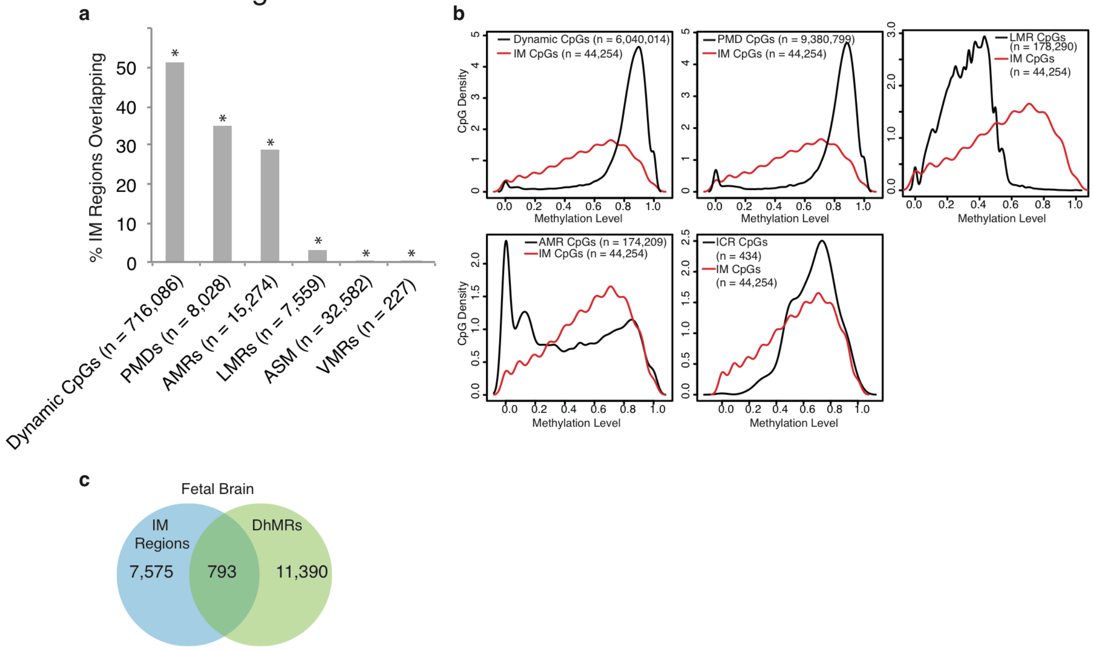


**Supplementary Figure 5. Comparison of IM to previous sets of variable methylation or ASM**

(a) Percentage of IM regions overlapping previously reported regions characterized by intermediate or dynamic tissue-specific methylation. AMRs=allelically methylated regions; ASM=allele-specific methylation; LMRs=lowly methylated regions; VMRs=variably methylated regions. (b) Distribution of CpG methylation levels from regions defined in previous studies as compared to IM. An Intermediate level of methylation, as assessed by WGBS in H1 ES cells, predominates within the IM regions reported in our manuscript and in imprinting control regions (ICRs), but is not a typical in regions previously reported as variably methylated. For each distribution shown, methylation levels were taken from the datasets used in the original manuscripts, or data from the same cell type as the original study. (a-b) *p<0.001, Chi-squared test. (c) Comparison of IM regions called in fetal brain samples to differentially hydroxymethylated regions (DhMRs), which show increased hydroxymethylation in fetal brain with respect to adult brain.

Extended Data Fig. 6

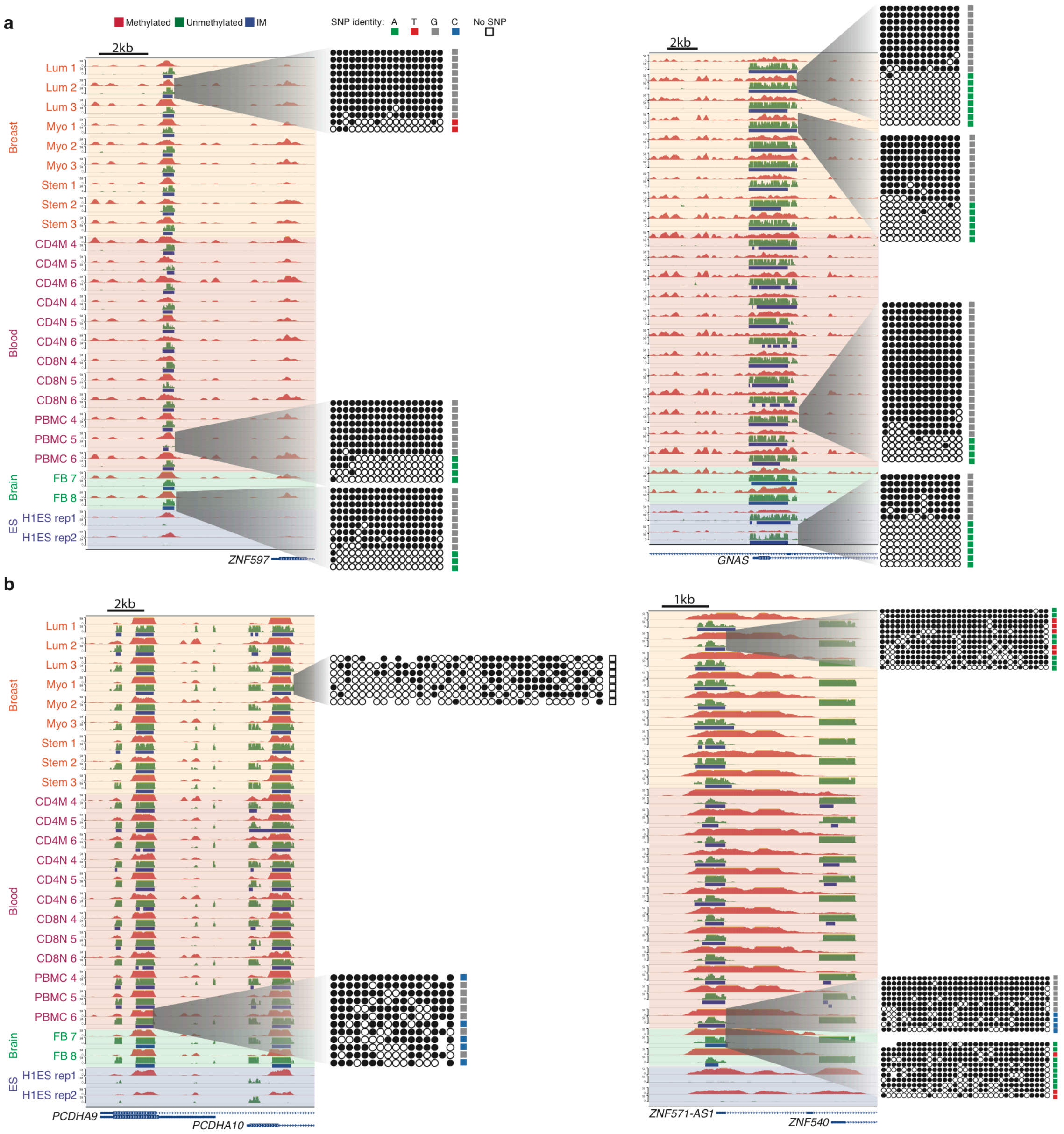


**Supplementary Figure 6. Validated ASM and AIM loci**

(a) ASM regions validated by targeted bisulfite PCR and sequencing. (b) AIM regions validated by targeted bisulfite PCR and sequencing. Height for all tracks shows a signal range from 0-50 reads.

## Supplementary Methods

### Enrichment Calculations

Enrichment based on methylation signal (methylated, unmethylated, or IM) over genomic features was calculated as a log odds ratio of the observed over expected proportions of CpGs within a particular feature. Note that restricting enrichment calculation to CpG overlap removes any bias based on the non-random distribution of CpGs across the genome. IM CpGs were those that fell within previously defined IM regions. Methylated CpGs were selected based on a read count of 4 or greater in MeDIP-Seq and no signal in MRE-Seq. Likewise, unmethylated CpGs were selected based on a read count of 4 or greater in MRE-Seq and no signal in MeDIP-Seq. The enrichment calculation is represented by the following formulae, using methylation as an example:

$$Enrichment = Log_2(Observed) - Log_2(Expected)$$

$$Observed = F_m/F_t$$

$$Expected = G_m/G_t$$

Where $F_m$ is the number of methylated CpGs within a feature, $F_t$ is the total number of CpGs within the feature, $G_m$ is the number of methylated CpGs in the genome, and $G_t$ is the total number of genomic CpGs.

## Supplementary References

1. Schulz, R. *et al.* WAMIDEX: a web atlas of murine genomic imprinting and differential expression. *Epigenetics* **3,** 89–96 (2008).

2. Ernst, J. & Kellis, M. ChromHMM: automating chromatin-state discovery and characterization. *Nat. Methods* **9,** 215–6 (2012).
